# *“I Always Buy the Purple Ones … If I See Them”:* Socioecological Factors Influencing Anthocyanin-Rich Food Consumption for Cognitive Health in Older Adults

**DOI:** 10.3390/nu15051194

**Published:** 2023-02-27

**Authors:** Katherine Kent, Karen Larsen-Truong, Catharine Fleming, Li Li, Lyn Phillipson, Genevieve Z. Steiner-Lim, Karen E. Charlton

**Affiliations:** 1 School of Medical Indigenous and Health Sciences, Faculty of Science Medicine and Health, University of Wollongong, Wollongong, NSW 2522, Australia; 2 School of Health Sciences, Western Sydney University, Penrith, NSW 2751, Australia; 3 Australian Health Services Research Institute, Faculty of Business, University of Wollongong, Wollongong, NSW 2522, Australia; 4 School of Science, Western Sydney University, Penrith, NSW 2751, Australia; 5 School of Health and Society, Faculty of the Arts Social Sciences and Humanities, University of Wollongong, Wollongong, NSW 2522, Australia; 6 NICM Health Research Institute, Western Sydney University, Penrith, NSW 2751, Australia; 7 Translational Health Research Institute, Western Sydney University, Penrith, NSW 2751, Australia

**Keywords:** anthocyanin, flavonoid, dietary strategy, behaviour change, qualitative, older adult, cognition, dementia, ageing

## Abstract

Despite the positive relationship between anthocyanin-rich foods and cognitive health, a dietary deficit exists in older adults. Effective interventions require an understanding of people’s dietary behaviors situated in social and cultural contexts. Therefore, the aim of this study was to explore older adults’ perceptions about increasing their consumption of anthocyanin-rich foods for cognitive health. Following an educational session and the provision of a recipe and information book, an online survey and focus groups with Australian adults aged 65 years or older (*n* = 20) explored the barriers and enablers towards eating more anthocyanin-rich foods and potential strategies to achieve dietary change. An iterative, qualitative analysis identified the themes and classified the barriers, enablers and strategies onto the Social-Ecological model levels of influence (individual, interpersonal, community, society). Enabling factors included a desire to eat healthily, taste preference and familiarity of anthocyanin-rich foods (individual), social support (community), and the availability of some anthocyanin-rich foods (society). The barriers included budget, dietary preferences and motivation (individual), household influences (interpersonal), limited availability and access to some anthocyanin-rich foods (community) and the cost and the seasonal variability (society). The strategies included increasing individual-level knowledge, skills, and confidence in utilizing anthocyanin-rich foods, educational initiatives about the potential cognitive benefits, and advocating to increase access to anthocyanin-rich foods in the food supply. This study provides for the first time, insight into the various levels of influence impacting older adults’ ability to consume an anthocyanin-rich diet for cognitive health. Future interventions should be tailored to reflect the barriers and enablers and to provide targeted education about anthocyanin-rich foods.

## 1. Introduction

Dementia was Australia’s second leading cause of mortality in 2019, accounting for 9.5% of all deaths [1]. In 2021, it was estimated that between 386,200 and 472,000 Australians were living with dementia, a figure that is projected to double by 2058 due to Australia’s ageing population [1]. Without a medical cure, identifying strategies that improve cognition and delay cognitive decline among older adults, both in Australia and internationally, is imperative to reduce the burden of dementia on individuals and society.

The role of nutrition for neuroprotection is a rapidly evolving area of research, and a group of bioactive compounds known as anthocyanins have been shown to display pro-cognitive properties in multiple populations. Anthocyanins are a subclass of dietary flavonoids that provide purple, red, and blue pigmentation in many plant-based foods [2,3]. Anthocyanins are numerous and wide spanning in nature, with over 1000 individual anthocyanins reported [4,5]. The major food sources of anthocyanins are berries such as blueberries and strawberries; fruits such as cherries and plums; vegetables such as red and black beans, red cabbage and onion; and beverages such as red wine, cranberry and red grape juice. Across Western countries, anthocyanin consumption has been estimated to be between 25 and 30 mg/day, and is influenced by cultural food choices, age, and gender [6]. A relative deficit in anthocyanin intake has been identified in older adults [7] and those living with dementia compared with younger adult populations [8,9], indicating a need for dietary support in these groups if the relevant health benefits of anthocyanins are to be conferred.

Preliminary evidence suggests that higher habitual consumption of anthocyanin-rich foods is associated with better memory in older adults with mild cognitive impairment (MCI) who are at high risk for developing dementia [10]. Human intervention studies have made significant headway investigating both the acute and longer-term cognitive effects of individual anthocyanin-rich foods in older people [11]. In particular, foods such as cherries [12], blueberries [13], and plums [14] have shown promise in improving cognition and vascular function (the latter of which is closely linked with dementia risk). For example, using a randomised controlled trial, Kent et al., demonstrated that the daily consumption of anthocyanin-rich cherry juice for 12 weeks improved measures of verbal learning and memory in older adults living with dementia [12]. However, the focus in clinical trials has been to utilize unique dietary interventions which deliver high-doses of anthocyanin-rich foods or supplements over short periods of time (e.g., 6–12 weeks), meaning that it is difficult to extrapolate their benefit to providing dietary guidance in real-world conditions. Further, there is a lack of consideration about the impact of whole-of-diet changes to increase anthocyanin intake for influencing cognitive function. Future clinical trials will aim to translate the current clinical trial literature towards examining the cognitive benefits of longer-term consumption of various anthocyanin-rich foods through dietary counselling [15]. Such efforts will focus on examining the long-term cognitive benefits of anthocyanin-rich dietary patterns, rather than examining the benefits of single food products or food-derived supplements.

While dietary approaches to managing neurodegenerative diseases have become increasingly important [16,17], older adults may struggle to adhere to a new diet that changes long-established patterns of eating behaviors or introduces new tastes [18]. This may be particularly challenging for older adults with cognitive decline, such as people with MCI and dementia [19]. Food choices and eating habits at an individual level are influenced by many interrelated factors, including various features of a person’s economic, social, and physical environment [20]. When adopting a fruit and vegetable-rich dietary pattern, often it is the higher cost or poor financial access to food that are identified as barriers to healthy eating in older people [21,22,23]. Availability of healthy food and physical access to food also influences eating patterns, with research showing that travelling longer distances to primary food stores reduces diet quality [24]. Other individual and interpersonal factors including social influences and individual motivations arising from the perceived health benefits of a diet can also play an important role in the eating behaviors of older people [25].

Despite some challenges in adopting a healthy fruit and vegetable-rich diet, older people consider these foods to be an integral part of a diet for healthy ageing [26] and tend to possess a more positive attitude towards functional foods than younger adults [27]. In particular, consumers can be strongly influenced by health claims related to disease prevention when choosing to eat functional foods [28]. Additionally, empowering older adults with an understanding of the health benefits of novel foods can support their consumption [29]. Correspondingly, older adults have shown to be able to adopt a fruit and vegetable-rich dietary pattern with a high level of adherence over short periods of time [30]. While older adults have self-reported that they could follow dietary changes over the longer-term, there are still substantial barriers and enablers to adopting dietary patterns for healthy ageing [23]. Much of the literature has focussed on establishing older adults’ perceptions of dietary patterns such as the Mediterranean diet [31] or the MIND diet [32]. However, research exploring perceptions of increasing consumption of a single-group of polyphenol-rich foods, such as purple anthocyanin-rich foods, is not yet available, but is required to inform future clinical trials.

As the potential barriers and enablers affecting older adults’ perceptions of eating more anthocyanins may be different to more comprehensive changes to total dietary patterns, and require different solutions, further elucidation of the barriers and enabling factors influencing the intake of anthocyanins for potential cognitive benefits is needed. This study aimed to explore the perceptions of older adults towards increasing their consumption of anthocyanin-rich food for cognitive health, and the perceived strategies that could enhance their consumption of these foods. The research question guiding this study was: “What are the barriers and enabling factors influencing anthocyanin-rich food intake in older adults, and what strategies do older adults perceive would support their consumption for cognitive health?”.

## 2. Materials and Methods

We carried out an educational session and qualitative study in December 2021 with approval from the Western Sydney University Human Research Ethics Committee (H14660), which was conducted in accordance with the principles of the Declaration of Helsinki [33].

### 2.1. Study Sample and Recruitment

This study recruited participants aged 65 years or older, who could speak English and lived in Australia. As anthocyanins are found in fruits and vegetables, people with dietary restrictions related to fruits and vegetables were not eligible to participate. A convenience sample of older adults was recruited purposively by circulating a flyer online, and by emailing the flyer to relevant community groups and health practitioners working with older adults who shared the flyer with their networks. Potential participants contacted the study team and were provided with a participant information sheet and consent form. Written informed consent was obtained from all participants. No eligible participants refused to participate or dropped out of the study.

### 2.2. Brief Educational Session

All participants (*n* = 20) attended a one-hour online information session delivered by a public health nutritionist (KK). The information session introduced the concept of anthocyanin-rich foods, discussed the types and sources of anthocyanin-rich foods available in the food supply, explained the connection between anthocyanins and cognitive function, and answered participant questions.

Following the educational session, a recipe and information book (Purple Food for Thought), developed by members of the research team (KK and KC), was sent to all participants via mail. The resource included an example shopping list for anthocyanin-rich foods (fresh, tinned and frozen), a food swap list that promoted anthocyanin-rich alternatives to common foods (e.g., encouraging participants to swap green grapes for purple grapes), and breakfast, savoury meal and snack-oriented recipes that contained anthocyanin-rich foods such as berries, cherries, eggplants, and purple carrots. Strategies to maintain anthocyanins in recipes were promoted through short cooking times and eating foods with minimal cooking and processing. A “What’s in Season” guide to anthocyanin-rich food was also included, in addition to identifying frozen and shelf-stable options such as black beans and frozen blueberries. Examples of some pages from the Purple Food for Thought book can be found in Supplementary Material. Participants were instructed to read and trial the recipes and engage with the associated material for a period of two weeks. There was no minimum or maximum quantity of anthocyanin-rich foods that participants were required to consume over this period of time, however participants were encouraged to eat more anthocyanin-rich foods generally.

### 2.3. Data Collection

A short online survey, developed for the purpose of this study, was emailed to all participants at the end of the two-week period which collected demographic information and asked three brief multiple-choice questions about food purchasing and cooking in the home.

All participants attended a one-hour Zoom (online) focus group facilitated by the lead researcher (KK) with support from other members of the research team (CF, LL and KL). Using a semi-structured guide, focus groups explored the participants’ perceptions and experiences of purchasing, cooking and consuming anthocyanin-rich foods, including the identification of barriers, enablers and potential strategies to support greater consumption. Example questions included: “What are some challenges that might prevent you from eating more anthocyanin-rich foods?” and “What in your life would make it easy for you to eat more anthocyanin-rich foods?”.

In addition to these discussions, participants were asked about their perceptions of the layout, attractiveness and usability of the “Purple Food for Thought” book. At the end of each focus group, participants were asked to comment on the potential barriers, enablers and strategies for a case study using a hypothetical character that had some cognitive impairment (developed by KLT, LP). These aspects of the focus group discussions provided data for a separate Design Thinking process that explored potential strategies to support recruitment, retention and adherence to an anthocyanin-rich diet in a clinical trial. Detail on this aspect of the focus groups and the associated results will be reported separately.

### 2.4. Analysis

Quantitative survey data were analysed using descriptive statistics, which was undertaken in SPSS Statistics for Windows, version 27 (SPSS Inc., Chicago, IL, USA).

Nine focus groups were conducted in total with an average recording time of 44 min per group (range 33 to 61 min). Recordings of each focus group were transcribed verbatim using Otter.ai (Los Altos, CA, USA) and transcripts were de-identified by coding responses with participant numbers. Transcripts were imported into NVivo for analysis (Version 10. QSR International).

We adopted an iterative analytical process based initially on Braun and Clarke’s six phases of thematic analysis [34]. An inductive, data-driven form of analysis was taken, where themes were constructed using latent coding, representing the researcher’s explicit interpretation of data. Thematic analysis was considered an appropriate approach for this study due to the capacity for rich description, sensitivity to context and to understand the experiences and perspectives of participants from an “emic” perspective [34]. Transcripts were read and re-read independently for immersion in the data. Initial codes were generated by one researcher (KK), and then the transcripts and codes were reviewed and revised by another researcher (KC). Braun and Clarke’s definition of a theme was used (i.e., stories about patterns of shared meaning across the dataset) throughout the analysis [34].

To answer our research question, the initial themes were subsequently categorised according to ‘barriers’, ‘enablers’ and ‘strategies’ to consuming anthocyanin-rich foods. Given that no new codes or themes were identified in the final focus groups that were coded, it was considered that additional data would not provide any new insights or information, suggesting that sufficient data had been collected. Experts have recommended [35] and we have previously shown [36] that a sample size of *n* = 18–20 is sufficient to achieve data saturation in exploratory qualitative research. Therefore, it was concluded that the point of data saturation had been reached [37] and no more participants were recruited. Once the initial themes were categorized, the identified barriers, enablers and strategies were mapped against the Social-Ecological Model to support further analysis and interpretation [38]. The model is a multilevel and interdisciplinary framework that allowed for barriers, enablers and strategies to be ordered according to four levels of influence: individual (intrapersonal), social networks (interpersonal), physical environmental (community) and macrosystem (society). Mapping the study findings to the Social-Ecological Model to guide data analysis facilitated an exploration of the multi-faceted influences on dietary behaviors and health outcomes, beyond individual decision-making, by identifying contextual factors (including cultural, historical, economic, and political factors) relevant to the supply and accessibility of anthocyanin-rich foods in the community. The final coding was reviewed and cross checked amongst the research team. To illustrate the themes, exemplar quotations were presented and referenced by participant number and age. Criteria in the COREQ, a 32-item checklist was utilised to report important aspects of the methodology [39].

## 3. Results

The sample of older adults (*n* = 20) had a mean age of 69 ± 2.8 years, ranging from 65 to 75 years, and just over half were female (60%; Table 1). Nearly half (45%) had a self-reported disability or health condition that impacted their daily life. More than a third of the sample (35%) had a postgraduate degree, 60% were retired and most (70%) were living with a partner and without dependent children at home (65%). The reported income was variable (Table 1). Half of the participants made the majority of their household food purchasing decisions, while more did the majority of the cooking (60%). Nearly all the participants shopped at major supermarkets (95%), but very few used home delivery or click-and-collect options to shop for food. Fruit and vegetable shops were frequented by three quarters of the participants (75%), and more than half consumed fresh produce grown from their garden (60%).

### 3.1. Barriers to Eating More Anthocyanin-Rich Foods for Cognitive Health in Older Australian Adults

The participants identified a range of barriers that they face to increase consumption of anthocyanin-rich purple foods to support cognitive health. Barriers were evident at the individual, interpersonal, community and society levels, as summarized in Table 2.

#### 3.1.1. Individual Level Influences

At an individual level, budgetary constraints, low motivation to cook complex recipes, personal food preferences, and existing food habits were identified as the barriers to adopting a diet with more anthocyanin-rich foods.

The “Purple Food for Thought” book provided participants with numerous suggestions around opportunities to increase the intake of anthocyanin-rich foods including food swaps or substitutions, such as swapping green apples for red apples. However, when discussing attempts to swap their regular purchases for more anthocyanin-rich foods, some participants mentioned the extra costs of anthocyanin-rich alternatives was a limiting factor within their food budget (Table 2).

*“the red cauliflower I bought yesterday compared to the white one was, you know, $2 or $3 more”* (Participant 14, age 71, Female).

Others reported that they were aware of the price difference but that this had not stopped them from buying these foods, but it would certainly be a consideration for other older adults on lower incomes.

*“So I’d be quite happy to buy a purple cabbage instead of a green cabbage but I do think [it] is expensive for people. And I think that purple sweet potatoes are ridiculously expensive compared to regular old sweet potatoes, but I don’t have to buy very many of them. So that’s fine. But I can see that some people would really struggle with it.”* (Participant 14, age 71, Female).

Most respondents reported being able to easily cook and consume anthocyanin-rich foods throughout the trial period. Others discussed that the effort required to cook and prepare some anthocyanin-rich recipes was prohibitive to them, as they preferred to cook more simple recipes:

*“Some of them [the anthocyanin-rich food recipes] are a little deceptive, I guess, in that it looks very simple, and then you start thinly slicing half a red cabbage and whatnot. And then realise, well, for me anyway, there’s a bit more work there.”* (Participant 18, age 69, Male).

Others reported they *“found the rigidity of the cooking and storage methods [suggested in the book to retain anthocyanins such as short cooking times and eating fresh food] a barrier”* to cooking and eating anthocyanin-rich foods. Some participants preferred to batch cook then freeze meals, or use *“long slow cooking, and freeze a lot of foods*” (Participant 5, age 72, Female) which were the methods that were suggested to avoid in the resource, as they are considered to degrade the anthocyanin-content in foods extensively. Occasionally, participants reported that some anthocyanin-rich foods were outside the realm of what they would typically cook:

*“I’m not an adventurous cook. I’m just a very bland cook.”* (Participant 17, age 71, Female).

Others disliked the taste of some of the common anthocyanin-rich foods. These factors were related to the participants’ food habits and the unfamiliarity of some anthocyanin-rich foods:

*“I have never cooked aubergine before”* (Participant 16, age 71, Male).

Even if some participants reported to be adventurous cooks and eaters, it was commonly reported that other people in their age group would be less flexible in adopting dietary changes.

*“I think the older you get, the less likely you are to want to do that. I just forget about my mom who’s 86. It’s trying to get her to eat anything different or consistently, she’ll try it for a little while, and then she gives up and you know, goes back to what she knows.”* (Participant 8, age 65, Female).

#### 3.1.2. Interpersonal Level Influences

Some participants expressed that their family and household food preferences was a limiting factor in their ability to buy and cook some anthocyanin-rich foods:

*“It probably limits me a little bit. Yeah. Yeah. Like for example, she [participant’s wife] doesn’t like eggplant. So you know, I’m doing an eggplant for me, then I have to do something else. So it’s not something I would do a lot.”* (Participant 15, age 75, Male).

#### 3.1.3. Community Level Influences

The most commonly reported barrier was the perceived limited availability of anthocyanin-rich foods in retail outlets where the participants usually shopped for food. While most of the participants reported that some purple foods were available, many identified that other anthocyanin-rich foods were difficult to find:

*“So availability became a major thing. I couldn’t get purple carrots at all. I couldn’t get some of the other ones like purple broccolini and potatoes. When I was able to get purple cauliflower, it only had some slightly tinted purple bits, so I wouldn’t say that had a lot of anthocyanin in [it] as a result. And purple asparagus, which we love and would eat, yes, none of those were readily available for us.”* (Participant 12, age 67, Female).

Sometimes, participants recalled wanting to purchase a particular anthocyanin-rich food but needing to travel to multiple shops, or try shops outside their usual, local stores to seek these foods.

*“I’m finding that getting these ingredients sometimes is very hard … like especially the fruit and, you know, the vegetables. I think [my husband] rang and we couldn’t find the purple sweet potato, anywhere. We went all around the area, even to an enormous fruit market … but they don’t carry any purple vegetables because there’s no demand.”* (Participant 17, age 71, Female).

#### 3.1.4. Society Level Influences

Anthocyanin-rich foods were commonly reported to be more expensive that other foods available in the shops, and were considered by some as being more “specialty goods”, compared with other fruits and vegetables.

*“So I bought pretty much as much as I could of the purple foods that were listed throughout in the cookbook. It wasn’t cheap, because they tend to be the specialty foods. And I ended up with a bill of 75 bucks.”* (Participant 9, age 67, Male).

The seasonality of many anthocyanin-rich fresh fruits and vegetables was a commonly reported barrier to consumption. The seasonality was perceived to influence both the availability and the cost of food. Data collection for this study occurred during the summer months when the seasonal berries and cherries were commonly available. However, participants frequently reported that many foods including vegetables, such as purple cauliflower and carrots, were not in season and were therefore unavailable. If foods were available outside of their peak season, they were often priced higher than other fresh fruits and vegetables.

*“When things are in season, they’re reasonably priced. When it’s not, then it can get quite expensive to buy a punnet of blueberries. Cherries you can only get once a year, you know, Australian cherries anyway.”* (Participant 8, age 65, Female).

Finally, participants often discussed that there was little society awareness regarding the health benefits of anthocyanin-rich foods, and there was little knowledge on a population-level of the potential benefits of anthocyanin-rich foods for cognitive health (Table 2).

*“Lots more education and general awareness is really needed about these purple foods”* (Participant 16, age 71, Male).

### 3.2. Enabling Factors towards Eating More Anthocyanin-Rich Food for Cognitive Health in Older Adults

The factors that could enable adoption of a more anthocyanin-rich diet were discussed and were, once again, mapped to the individual, interpersonal and community levels of the Social-Ecological Model.

#### 3.2.1. Individual Level Influences

At the individual level, health consciousness, familiarity with purple foods, a preference for purple foods and the ability to grow your own were seen as enablers. Some participants reported that they were motivated to consume anthocyanin-rich foods for health reasons.

*“I actually had known that, that purple foods were beneficial, and had sort of made myself a bit of a commitment… And I want to keep it up because I just decided for myself, that I will have something with purple food every day. And I think that’s possible for me...”* (Participant 11, age 71, Female).

Most participants reported liking the taste of many anthocyanin-rich foods, which supported their consumption of these foods. Many participants sought out purple varietals of the common fruits and vegetables they consumed:

*“I really like Brussel sprouts. And I always buy the purple ones if I see them. So, and I often see the purple cauliflower … and I love cauliflower. Oh, goody. There it is. I’ll add that.”* (Participant 14, age 71, Female).

Participants discussed that there were some anthocyanin-rich foods that they consumed regularly, and this familiarity was considered an enabling factor that supported their possible adoption of an anthocyanin-rich diet.

*“I changed over to red onion probably over 12 months ago. So that’s for no other reason than I preferred it.”* (Participant 1, age 71, Female).

A minority of participants discussed that they enjoyed growing food, and had experience growing some rarer purple foods, which supported their consumption of them (Table 3).

#### 3.2.2. Interpersonal Level Influences

Social support was an important enabling factor that was identified by participants. Some participants discussed that they had shared their understanding of the benefits of purple foods with other older adults in their social networks:

*“People have been interested in [hearing about purple foods], you know, friends I’ve been around with, it’s something to talk about that’s not just gossip”* (Participant 8, age 65, Female).

Participants also discussed that their friends were also motivated to eat purple foods if there were health benefits, which would support their consumption of anthocyanin-rich foods (Table 3).

#### 3.2.3. Community Level Influences

While most participants believed that the availability of foods was a concern for them, others did not have trouble accessing some anthocyanin-rich food, reporting that many purple foods were available from major supermarkets and markets:

*“I’m lucky in that I have access to a farmers market.”* (Participant 7, age 73, Female).

Some were of the impression that if some anthocyanin-rich foods weren’t available, others would be:

*“I believe there will always be a substitute, you know, there, there may not be, say the eggplant but you look around, you’ll see a different one that is still purple … all year round. That’s my belief, even if it’s red onions.”* (Participant 6, age 70, Female).

Others reported that the availability of frozen foods was able to help them to continue consuming anthocyanin-rich foods:

*“Like with the blueberries … we can get those easily from Woolworths [major supermarket] … And so are all the other lovely frozen fruits. Easy peasy.”* (Participant 17, age 71, Female).

### 3.3. Strategies to Support Consumption of Anthocyanin-Rich Foods for Older Adults

Participants identified a range of potential strategies that could support them to eat more anthocyanin-rich foods. Table 4 provides a summary of the strategies that were mapped to the individual, community and society levels. Participants did not identify any interpersonal strategies.

#### 3.3.1. Individual Level Strategies

The individual-level barriers included low motivation for cooking in some participants and the lack of familiarity with a variety of anthocyanin-rich fruits and vegetables (Table 3), and therefore participants discussed that strategies to increase consumption at an individual level should focus on dietary changes that are as simple as possible (Table 4). As an example, food swaps (where a low anthocyanin food is swapped for an anthocyanin-rich food) were reported by most participants to be the most feasible and achievable way to increase the amount of anthocyanins within their diet:

*“Just buy the red cabbage instead of the, you know, the green, for the coleslaw.”* (Participant 8, age 65, Female).

Some participants thought that developing strategies for people to fit anthocyanins within their usual meal options would support them to consume these foods:

*“As a dedicated cannibal [it] would be really helpful to have a bunch of stuff that I could fit around my protein portion.”* (Participant 10, age 67, Male).

Education and individualised support from nutrition professionals to help people increase their consumption of purple foods was discussed by a minority of participants. Education was thought to include what foods contain anthocyanins, where to source them and how to prepare them:

*“Maybe letting people know not to be afraid of frozen foods. Yeah. And if there is a problem that a budget issue then to check out those, those [cheaper] options.”* (Participant 11, age 71, Female).

#### 3.3.2. Community Level Strategies

Participants felt that community-level education initiatives about anthocyanin-rich foods, including recipe demonstrations, would be well received by older adults (Table 4).

*“You could go to Probus [Australian Retirement Club] meetings, they’re always talking, you know, about new things that come up, to educate us. I think there’s a magazine that comes out.”* (Participant 6, age 70, Female).

The social support of community education was seen to be particularly influential.

*“Sharing ideas is really powerful. I mean, we were involved with a few little groups”* (Participant 15, age 75, Male).

Additionally, some suggested that point-of-purchase advertising in shops could be a suitable way to prompt older adults to purchase anthocyanin-rich foods.

*“Have you thought about advertising or putting up posters in a supermarket?”* (Participant 4, age 65, Female).

#### 3.3.3. Society Level Strategies

Participants suggested that broad education should be provided to both older adults and younger people that highlights the potential cognitive benefits of anthocyanin-rich foods, including through media and print publications (Table 4). Some discussed that eating more purple food within a daily diet should be ‘normalised’ across the Australian population.

*“Let’s have school kids growing purple carrots in their school garden, teenagers thinking purple sweet potato [is] cool and families repeatedly colouring their meals purple. By the time they reach 65, purple foods will be normalised in their diets as a wholesome way forward.”* (Participant 9, age 67, Male).

Participants also thought that more could be done to advocate for purple foods and raise awareness of the research being conducted, *“I don’t think there’s enough publicity out there for the value of the of the purple foods”* (Participant 16, age 71, Male). It was also suggested by some participants that celebrity endorsement and high-profile media stories would be beneficial for increasing consumption of anthocyanin-rich foods:

*“You need someone like a ‘Fast Ed’ or a ‘Karen Martini’ [both Australian celebrity chefs] who would take that message on board and simplify it”* (Participant 20, age 68, Female).

To overcome the issues with the lower availability of purple foods, some participants suggested that industry support from both *“growers associations”* and *“Coles or Woolworths”* [major supermarkets] could be further harnessed to both promote anthocyanin-rich foods and support the continued availability of anthocyanin-rich foods across Australia (Table 4):

*“What I’d like to do is talk with the growers, and simply say, “Are they aware?” [of the potential cognitive benefits of anthocyanin-rich food]. I would like to think they could probably cultivate a market, and I think there’s enough people that would be really interested in trying and experimenting.”* (Participant 9, age 67, Male).

## 4. Discussion

Through the provision of a brief educational session and subsequent focus groups, this study explored the barriers and enablers that influence older adults when increasing their consumption of anthocyanin-rich foods for cognitive health, and future strategies that could support dietary changes in this group. Using the Social-Ecological Model, this study made connections between an individuals’ lived experiences, and demonstrated how their networks, community and wider food systems influence their decision-making abilities and dietary behaviors.

The older adults in this study suggested that a substantial number of factors influence their ability to make dietary changes. Individual food and cooking preferences were identified as both a key barrier and enabling factor. Supporting this finding, older adults who report liking vegetables are more likely to consume a higher quantity and variety of these foods [40]. In our study, the participants enjoyed eating some familiar anthocyanin-rich foods, but others were considered out of the ordinary for what they would usually purchase and consume. The participants expressed an interest in trying some new purple foods, but they considered that in general, older adults, especially those who are older than the current participants, might be reluctant to include unfamiliar foods rich in anthocyanins in their diets. This perception is supported by consumer research showing that older adults’ acceptability to consume novel foods can be influenced by multiple factors including education, gender and food motives [41]. Older adults, like other groups of consumers, can be either enthusiastic or resistant to trying novel fruits and vegetables, and older males can be particularly resistant to making dietary changes to incorporate unfamiliar foods [42,43]. Factors such as healthy food knowledge can influence an individual’s intention to eat a healthy diet [44]. In our study, some older adults spoke about different preferences within their household and the effect that this had on making changes to their diet. In a study by Choi et al., 2020, social support was shown to influence fruit and vegetable consumption in older adults [45]. Additionally, as also seen in our study, the family influence is a primary enabling factor towards healthy dietary habits [46] including in older adults [31]. Together, these findings highlight the highly individual nature of food preferences and behaviors and are in line with other research related to introducing novel foods to an individual’s diet. 

Taste appeared to be an important consideration that supported the consumption of some anthocyanin-rich foods, and this finding is supported by other research on the influence of taste on food choices in people across the lifespan [47]. Taste preferences and ‘an appetite’ for fruit and vegetables, has been reported to be among the top factors impacting older adults ability to achieve dietary recommendations for these foods [48]. Further, taste preferences have been previously identified as a barrier and enabler for dietary change or the adoption of a fruit and vegetable rich Mediterranean diet [23,32,49,50,51]. Similar to other research on fruits and vegetables, our participants maintained a preference toward familiar meals and simple recipes that avoided complexity in purchasing, organizing, and preparing food [52].

It has been reported that consumers value food choices that maintain cultural heritage, underscoring the importance of supporting the consumption of traditional foods [53]. Further, consumers that value traditional eating are more likely to possess an intention towards adopting a healthy diet [54]. In support of this, in our study, the participant’s suggestions for strategies to promote a more anthocyanin-rich diet focused on simple educational strategies that were easy for older adults to implement, including small changes or tweaks to familiar or traditional recipes. Therefore, interventions using nutrition education materials such as traditional recipes, meal plans, shopping lists and food exchange lists to amend recipes could support diet adherence in older adults [55].

At a community level, participants highlighted the important influence of the availability of anthocyanin-rich foods. This finding may have been influenced by the supply chain issues resulting from the ongoing COVID-19 pandemic in Australia, even though the participants were not in a lock-down at the time of the study [56,57]. Seasonality was considered to influence the availability of anthocyanin-rich food substantially, especially for some summer fruits such as berries and cherries, and winter vegetables such as purple cauliflower and carrots. This is aligned with other research showing that the seasonality of fruits and vegetables is a leading factor influencing the purchase and consumption of these foods [58]. Some research has found a reluctance of some consumers towards limiting food choices to only what is in season [59,60]. However, potentially enhancing the promotion and availability of seasonal and locally-grown foods within communities may help overcome barriers to their consumption [53].The occasional comments about utilizing frozen fruit alternatives may indicate the need to highlight the role of non-fresh and non-perishable options to increase anthocyanin intake among older adults, particular when availability and physical and/or economic access to food is of concern.

A frequently mentioned barrier identified in our study was the cost of anthocyanin-rich foods. Several participants noted that they were not personally affected but suggested that this would likely be a strong barrier for pensioners and older adults with lower incomes. The cost of food has been shown to be a significant factor in people’s choice of food and consumption [61]. Indeed, recently published Australian research has identified cost as a major barrier to consuming sufficient fruits and vegetables in a diverse sample of middle to older aged adults [62], as well as older adults on low incomes [63]. As the cost of food, and healthy food in particular, continues to rise following the COVID-19 pandemic in Australia [64], interventions that reduce the cost of food are likely to support healthy food choices in older adults. In our study, and in the broader literature, perception of the price of fruits and vegetables is linked to their seasonality, with in-season produce perceived as cheaper [65]. The perceived higher cost of anthocyanin-rich foods could be a threat to the long-term adoption of an anthocyanins-rich diet, and is consistent with previous research related to the adoption of a Mediterranean-style diet [31] and other novel foods [41].

The availability and process of physically procuring anthocyanin-rich foods was an important barrier experienced by some participants, but was conversely identified as an enabler by others. While some participants reported already growing purple food at home and that they had easy access to farmers markets, others reported travelling outside their neighborhoods during the two-week study period to seek anthocyanin-rich foods. The challenges that older people face in procuring food in urban environments has been identified by other authors, along with adaptive behaviors such as making trips to multiple stores to obtain the quality of foods preferred at prices that fit within limited food budgets [66]. While these adaptive behaviors, some of which were demonstrated by our participants, are commendable for our short-term study they do present a significant barrier to adoption of an anthocyanin-rich diet over the longer-term, especially for older adults without reliable transportation [67]. Interventions that support access to healthy food in addition to addressing transportation needs for older adults to access healthy food retailers may be needed in some communities.

When considering strategies to support the increase in consumption of anthocyanin-rich purple foods for cognitive health, participants in our study reflected mostly upon strategies that would impact the community and society level factors. This is in contrast to many of the commonly developed strategies that are implemented to support dietary behavior change and that largely focus on developing personal skills [68]. The “Purple Food for Thought” book developed for this study, for instance, provides mostly individual-level education towards making dietary changes. While still an important consideration, this study’s findings suggest that interventions and programs aimed at supporting older adults’ consumption of anthocyanin-rich foods could benefit from strategies that create supportive environments and involve the community [69].

Population-level messaging could be effective at raising the profile of anthocyanin-rich foods and normalizing their consumption. For instance, our participants suggested that targeted education initiatives could be provided through community networks and point-of-purchase food prompts within supermarkets, at checkouts or in advertising material associated with food shopping. Such community-led education initiatives have been shown to be effective in increasing the knowledge of fruits and vegetables in older adult populations [70], including the potential health benefits of phytochemicals [71]. The sustained, well-executed marketing of fruits and vegetables has been shown to be effective in improving nutrition knowledge, attitudes and consumption behaviors, including for polyphenol-rich dietary patterns like the Mediterranean diet [72], but this requires substantial investment [73,74]. Some participants suggested that older adults might ‘buy in’ to messaging about anthocyanin-rich foods if it was promoted by a celebrity chef (or similar). Some research reports that media is a key source of nutrition information for the general public [75]. However, other research from the UK has suggested that this is not an effective strategy in changing behavior as celebrity chefs are seen as entertainers rather than as a reliable source of health advice [76]. Regardless, this approach should be further tested in the Australian context and with older adults specifically.

At a society level, advocating for initiatives that could extend the seasonal availability and associated price fluctuations of commonly consumed anthocyanin-rich foods was offered as a potential strategy. A recent review has suggested that subsidizing the cost of fruits and vegetables by 10% is associated with a 14% increase in their consumption [77]. However, overcoming the seasonal variability of anthocyanin-rich foods may be more challenging, especially in light of arguments that call for the re-localization of food supply systems to address sustainability concerns of global food supplies [78,79]. Instead, older adults should be supported to identify and consume anthocyanin-rich seasonal produce. To support a year-round anthocyanin-rich diet, promoting the consumption of foods with longer-shelf lives such as frozen berries and fruits, canned foods, vacuum packed, and shelf-stable juices could complement seasonally available foods, and support the habitual consumption of anthocyanin-rich foods for cognitive health in older adults. Further development of storage and packaging technologies that would prolong shelf-life and maintain the anthocyanin content and sensory qualities of fruits and vegetables may also help sustain a diet rich in anthocyanins [80,81].

### Strengths and Limitations

To our knowledge, this study is the first to explore barriers and enablers to adopting an anthocyanin-rich diet for cognitive health, and the first study to use the Social-Ecological Model to investigate the various levels of influence impacting older adults when making dietary changes for specific polyphenols, in this case anthocyanin-rich foods. The findings of this research contribute to the evidence-base related to understanding the feasibility of dietary changes to support cognitive health. The results are useful for informing the design of dietary strategies and the level of support required to enable older adults to make dietary changes in future research. The use of focus groups permitted insights into the barriers and enablers that affect dietary behaviors in older adults, however the risk of bias in terms of an individual’s desire to conform to social acceptability cannot be ruled out [82].

Our study used a purposive sampling strategy to open recruitment to a broad range of individuals from the general population. Given this strategy, controlling for factors such as cultural background, gender, age, and years of experience was not possible and not within the scope of this project. Our non-representative sample, of mostly well-educated and higher-income individuals may have resulted in recruitment bias, possibly leading to a skewed group of older adults who are more health conscious. It is possible that the subjective experiences of our sample may not be representative of a larger population. In future, purposive sampling of older adults from different demographic and cultural groups could provide a broader understanding of the barriers and enablers affecting older adults across Australia and other countries. While comparative analysis was not a focus of this exploratory, qualitative study, future research could extend our findings by comparing the experience of older adults from other demographics, and exploring variations between cultural groups, as differences have been reported between consumer perceptions of healthy diets between countries [54,83].

## 5. Conclusions

In conclusion, our results suggest that older adults are faced with a range of barriers and enabling factors when attempting to increase their consumption of anthocyanin-rich foods for cognitive health. Individual and household preferences, established habits and behaviours, and the availability of anthocyanin-rich foods are important influences on older adults’ abilities to adopt an anthocyanin-rich diet for cognitive health. Health consciousness and social support may support older adults to make dietary changes, but low motivation for complex cooking methods and the introduction of novel or non-traditional foods may be barriers to adopting an anthocyanin-rich diet.

Our findings are relevant for multiple stakeholders. For nutrition professionals, our results show that dietary messaging should be tailored to address the common barriers identified in this age group such as cooking motivation and overcoming seasonality. Further, nutrition researchers should also consider the substantial influences that can impact on dietary behaviour change for anthocyanin-rich food when designing studies. Consideration of these factors should be used to inform the design of clinical trials and resources to support dietary change that could support older adults in eating more anthocyanin-rich foods.

Given the need to translate clinical trial outcomes into practical dietary messaging, our results suggest that targeted education about the benefits of anthocyanin-rich foods for cognitive health could be provided through community networks and population-level messaging could be effective at raising the profile of anthocyanin-rich foods. For food supply stakeholders, our results suggest that providing point-of-purchase food prompts and supporting initiatives that extend the seasonal availability and reduce the cost of familiar and commonly consumed anthocyanin-rich foods could reduce the societal level influences. Future research into the predictors of anthocyanin-food purchasing and consumption behaviours, in addition to the more objective measures of individual, interpersonal, community and society level factors could help identify clearer opportunities for interventions to support greater anthocyanin-rich food consumption in older adults.

## Figures and Tables

**Table 1:** Overview of the study sample demographic characteristics and food shopping and cooking habits.

Characteristic	Category	Descriptive Statistic
Age, years mean ± SD		69.3 ± 2.8
Gender *n* (%)	Male	6 (30%)
	Female	14 (70%)
Has a health condition or disability that limits activity	Yes a little	9 (45%)
	Yes a lot	0 (0%)
	No	11 (55%)
Highest level of education *n* (%)	Postgraduate degree	7 (35%)
	Bachelor’s degree	4 (20%)
	TAFE certificate or diploma (or equivalent)	4 (20%)
	Secondary education–completed year 12	2 (10%)
	Secondary education–completed year 10	3 (15%)
Relationship status	Partnered, married or defacto, living together	14 (70%)
	Single, never married	1 (5%)
	Single (widowed, divorced or separated)	4 (20%)
	Partnered, married or defacto, living apart	1 (5%)
Household composition	Couple with no dependent children at home	13 (65%)
	Single person with no dependent children at home	5 (5%)
	Couple with dependent children at home	1 (5%)
	Single person with dependent children at home	1 (5%)
Occupation status	Employed	6 (30%)
	Retired	12 (60%)
	Homemaker/family carer	2 (10%)
Income	I don’t know or would prefer not to answer	3 (15%)
	$20,000–$60,000	5 (25%)
	$60,000–$100,000	5 (25%)
	$100,000–$150,000	4 (20%)
	$150,000+	3 (15%)
Makes the majority of food purchasing decisions for the household	Yes	10 (50%)
	No	1 (5%)
	It is equal with a partner	9 (45%)
Does the majority of cooking in the household	Yes	12 (60%)
	No	4 (20%)
	It is equal with a partner	2 (20%)
Source of food purchased over the previous month	Major Supermarket (instore)	19 (95%)
	Independent Supermarket (instore)	10 (50%)
	Home delivery of groceries	0 (0%)
	Click and collect groceries	1 (5%)
	Fruit and Vegetable Shop (instore)	15 (75%)
	Restaurants/Cafes (instore)	10 (50%)
	Restaurants/Cafes (takeaway)	3 (15%)
	Own garden	12 (60%)

**Table 2:** Key barriers to increasing consumption of anthocyanin-rich purple foods for cognitive health in older Australian adults.

Subthemes Mapped to the Components of the Social-Ecological Model		Definition	Exemplifying Quotation
Individual level influences	Food budget constraints	The cost of some anthocyanin-rich purple foods was prohibitive.	*We have been getting purple food for quite a while. And, yes, we’re just very limited on what you can sort of get price wise (Participant 3, age 71, Female).*
	Cooking motivation	Some people preferred to avoid cooking complicated recipes.	*I’m just a lazy cook. Now. I just do as little as possible when it comes to cooking (Participant 17, age 71, Female).*
	Food preferences	Some anthocyanin-rich foods were disliked.	*I’m not a big fan of juices, I didn’t buy it (Participant 20, age 68, Female).*
	Food habits and familiarity of foods	Some participants preferred to eat their typical diet which might preclude some unfamiliar anthocyanin-rich foods.	*I think a lot of people in this age group will not be familiar with a number of the purple ingredients (Participant 12, age 67, Female).*
Interpersonal level influences	Household composition	Cooking for a household sometimes influenced a person’s ability to choose purple foods.	*There are some [anthocyanin-rich foods] that she [my wife] doesn’t like but that I do (Participant 15, age 75, Male).*
Community level influences	Limited availability of some anthocyanin-rich foods	The lack of some anthocyanin-rich purple foods in the shops limited participants ability to buy and consume these foods	*The biggest challenge is finding the purple foods in the local supermarket. (Participant 12, age 67, Female).*
Society level influences	Higher price of purple foods	Negative impact of perceived higher cost of purple anthocyanin-rich foods	*A purple cabbage is more expensive than green cabbage. So for … people who are worried about their budget, then they may think well, no, I’ll just do the green cabbage because it’s cheaper (Participant 14, age 71, Female).*
	Seasonal variations in food supply	Related to availability and cost. the seasonality of foods was perceived to influence a person’s ability to buy and consume these foods.	*When things are in season, they’re reasonably priced. When it’s not, then it can get quite expensive to buy a punnet of blueberries (Participant 8, age 65, Female).*
	Limited promotion of anthocyanin-rich foods	Some participants felt that there was insufficient promotion of anthocyanin-rich foods, including at point-of-purchase	*I don’t think there’s enough publicity out there for the value of the of the purple foods (Participant 6, age 70, Female).*

**Table 3:** Key enabling factors for increasing consumption of anthocyanin-rich purple foods for cognitive health in older Australian adults.

Subthemes Mapped to the Components of the Social-Ecological Model		Definition	Exemplifying Quotation
Individual level influences	Health consciousness	A perception of some participants that makes healthy eating desirable	*I’m motivated around diet. Sure. I mean, I have my lapses. Christmas slips? Yes. (Participant 1, age 71, Female).*
	Food preferences	Favouring the taste of anthocyanin-rich foods	*[regarding purple cabbage] I actually love it ... In fact, it tastes better than the regular kind. (Participant 15, age 75, Male).*
	Familiarity of foods	Familiar purple foods were commonly consumed by participants	*I eat a lot of purple fruits, normally my diet. (Participant 2, age 65, Male).*
	Gardening	Growing purple foods at home was an enabling factor for some participants	*I have been growing purple carrots for some years now. (Participant 12, age 67, Female).*
Interpersonal level influences	Support from social networks	Sharing knowledge with social networks about anthocyanin-rich foods was thought to positively influence their consumption	*We have many friends who will enthusiastically embrace eating foods with health benefits … they are keen to try purple foods, particularly when they hear of the great research being carried out. (Participant 16, age, 71, Male).*
Community level influences	Availability	Some anthocyanin-rich foods were perceived to be readily available in local shops and farmers markets.	*There is the frozen option but at the moment the fruit is absolutely beautiful because it’s at its peak. But when it isn’t, the frozen makes it very easy. (Participant 13, age 70, Female).*
Society level influences	-	-	-

**Table 4:** Key strategies to promote enablers and reduce barriers towards increasing consumption of anthocyanin-rich purple foods for cognitive health in older Australian adults.

Subthemes Mapped to the Components of the Social-Ecological Model		Definition	Exemplifying Quotation
Individual level	Simple dietary changes	Easy changes to increase consumption of anthocyanin-rich food, such as food swaps, were considered to be most successful.	*I think making it easy making it [eating more purple foods] convenient and quick. (Participant 20, age 68, Female)*
	Individual-level education	Increasing education and support provided at an individual level would be helpful towards increasing consumption of purple foods	*Lots more education and general awareness is really needed about these purple foods… That’s what I needed. The education. (Participant 17, age 71, Female).*
Interpersonal level	-	-	*-*
Community level	Community-level education	Education through community groups and at points-of-purchase could support older adults to eat purple foods	*The only thing would be to have a little demonstration and serve up some food. You know, because there are a lot of retirement type villages and gated communities now that have halls and rooms that, you know, this could be set up in. (Participant 17, age 71, Female).*
Society level	Raise profile of purple foods	Bringing more attention to purple foods in popular media was thought to be a way to increase awareness of these foods	*Probably raising awareness in the media would be another thing, because you don’t… Well, I haven’t seen a whole lot of stories, I see the odd story that comes up, mostly about blueberries. (Participant 15, age 75, Male).*
	Population level education	Education across the lifespan (not just for older adults) was thought to be important for normalising purple foods.	*More education targeted to 65 and over …that’s got to be big television or magazines, I would think (Participant 5, age 72, Female).*
	Gain industry support	Industry support from across the food supply chain was seen as critical towards increasing the supply and availability of purple foods	*We have to bring the food industry in on this. And I’m sure if you can get that groundswell … you’ve just got to look at what’s available now in terms of high quality dietary food in supermarkets as compared with five years ago. They’re sensitive to those sorts of shifts. (Participant 9, age 67, Male).*

## Data Availability

The data that support the findings of this study are available from the corresponding author, K.K., upon reasonable request.

## Supplementary Materials

The following supporting information can be downloaded at: https://www.mdpi.com/article/10.3390/nu15051194/s1, File S1: Examples of some pages from the “Purple Food for Thought” book can be found in Supplementary Material.
