# Supplementary material for: “I Always Buy the Purple Ones … If I See Them”: Socioecological Factors Influencing Anthocyanin-Rich Food Consumption for Cognitive Health in Older Adults
Source: Nutrients. 2023 Feb 27;15(5):1194. doi: 10.3390/nu15051194 (PMC10005612; doi:10.3390/nu15051194)

VERY BERRY SMOOTHIE

SERVES 1 - LOW ANTHOCYANIN

INGREDIENTS

- ½ cup frozen blueberries (60g)
- ½ medium banana (50g)
- 1 tablespoon nut butter
    - e.g. peanut, almond (25g)
- 1 cup milk of choice e.g. dairy, soy (250ml)
- ½ cup high protein yogurt (130g)
- Ice

DIRECTIONS

1. Place all ingredients in high speed blender
2. Blend until smooth
3. Pour smoothie into glass and enjoy

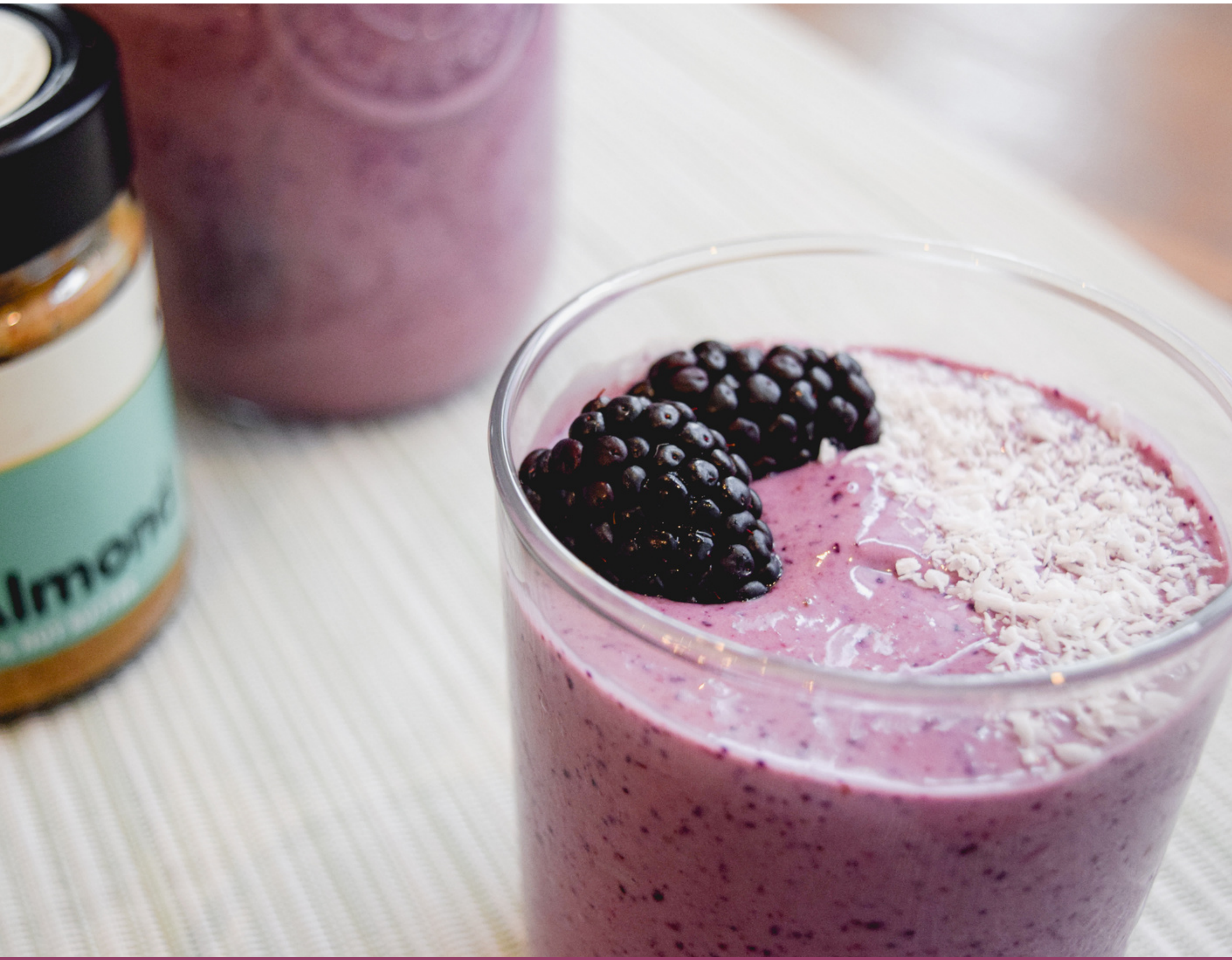

Gently steam or roast purple veggies including eggplant, purple kale, purple potatoes, purple cauliflower, radicchio and red onion with a glug of olive oil. A quick cooking time leaves them crisp and tender, and retains more anthocyanins.

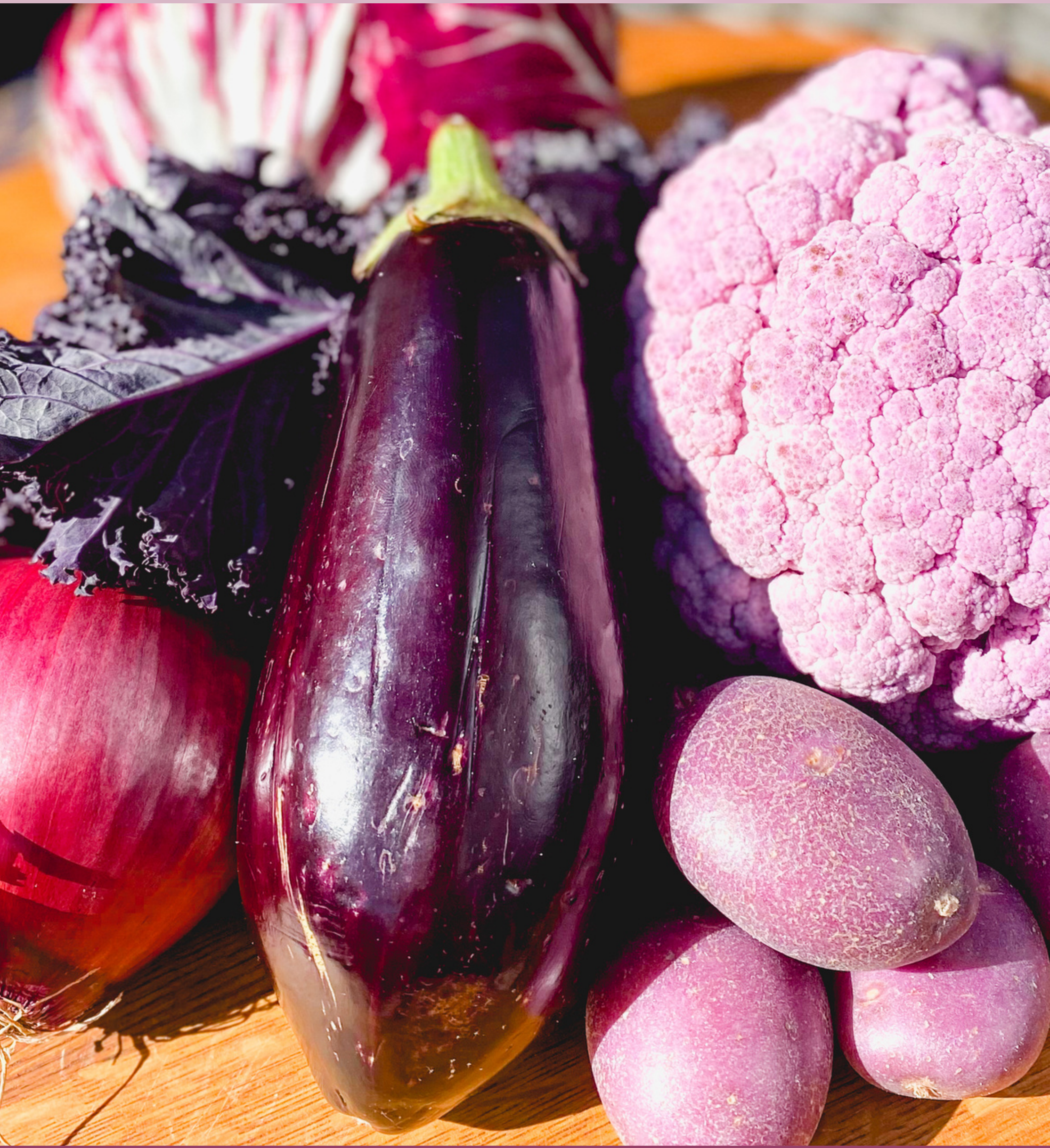

SIMPLE FOOD SWAPS TO INCREASE THE ANTHOCYANINS IN YOUR FAVOURITE MEALS

Brown onions	Red onions
Green grapes	Red & Purple grapes
Green Cabbage	Purple Cabbage
White wine	Red wine
White rice	Black rice
Iceberg Lettuce	Red leaf lettuce
Cauliflower	Purple Cauliflower
Green apples	Red apples
Marmalade	Berry jams
Orange Juice	Blood orange juice
Chickpeas	Blackbeans
White vinegar	Red wine vinegar

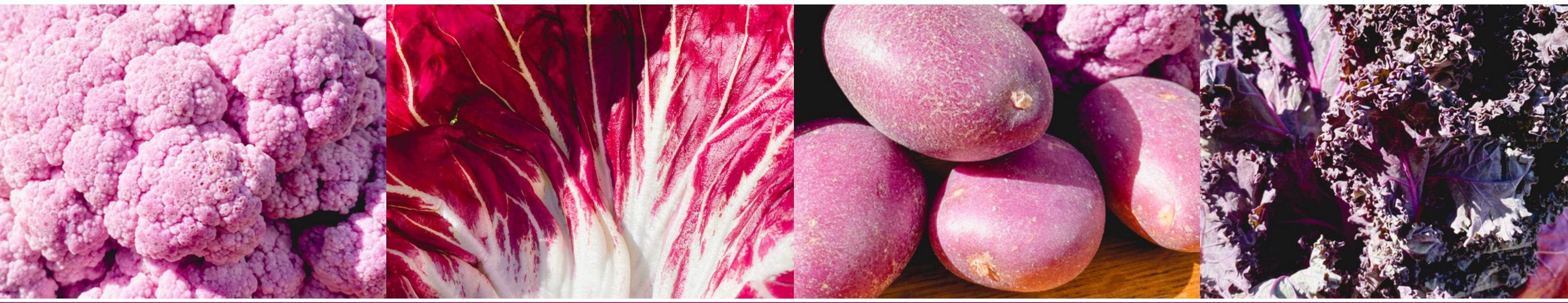

WHAT'S IN SEASON FOR ANTHOCYANINS?

SUMMER

FRUIT

- Blackberry
- Blueberry
- Boysenberry
- Cherry
- Currant
- Guava
- Mulberry
- Raspberry
- Rhubarb
- Strawberry

VEGETABLES

- Purple asparagus
- Eggplant
- Radish

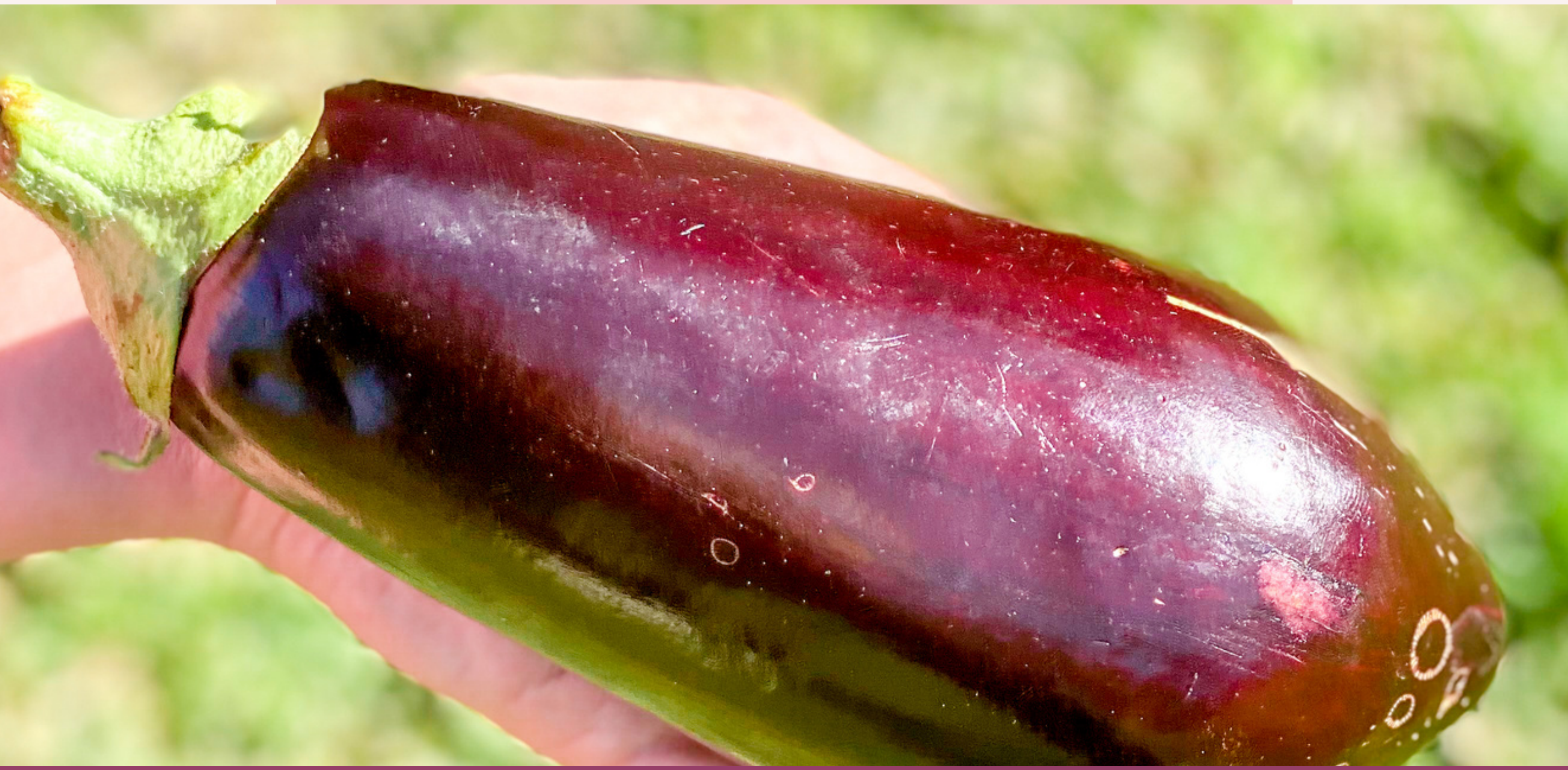

Supplement: Supplementary file 1 [file nutrients-15-01194-s001.zip › nutrients-2212532-supplementary.pdf]
